# Comparison of cytokines levels among COVID-19 patients living at sea level and high altitude

**DOI:** 10.1186/s12879-022-07079-x

**Published:** 2022-01-28

**Authors:** Juana del Valle-Mendoza, Yordi Tarazona-Castro, Alfredo Merino-Luna, Hugo Carrillo-Ng, Sungmin Kym, Miguel Angel Aguilar-Luis, Luis J. del Valle, Ronald Aquino-Ortega, Johanna Martins-Luna, Isaac Peña-Tuesta, Wilmer Silva-Caso

**Affiliations:** 1 School of Medicine, Research Center of the Faculty of Health Sciences, Universidad Peruana de Ciencias Aplicadas, Lima, Peru; 2 Laboratorio de Biologia Molecular, Instituto de Investigación Nutricional, Lima, Peru; 3 Unidad de Cuidados Intensivos, Clinica San Pablo, Sede Huaraz, Huarza, Peru; 4 Korea International Cooperation for Infectious Diseases, Chungnam National University College of Medicine, Daejeon, South Korea; 5 Barcelona Research Center for Multiscale Science and Engineering, Departament d’Enginyeria Química, EEBE, Universitat Politècnica de Catalunya (UPC), Barcelona, Spain; 6 Universidad Peruana de Ciencias Aplicadas, Av. San Marcos Cuadra 2, Chorrillos, Lima, Peru

**Keywords:** COVID-19, SARS-CoV-2, Cytokines, Different altitudes, Peru

## Abstract

**Background:**

At the end of 2019, a novel coronavirus denominated SARS-CoV-2 rapidly spread through the world causing the pandemic coronavirus disease known as COVID-19. The difference in the inflammatory response against SARS-CoV-2 infection among people living at different altitudes is a variable not yet studied.

**Methods:**

A descriptive cross-sectional study was performed in two Peruvian cities at different altitudes for comparison: Lima and Huaraz. Five important proinflammatory cytokines were measured including: IL-6, IL-2, IL-10, IFN-**γ** and TNF-α using ELISA assays.

**Results:**

A total of 35 COVID-19 patients and 10 healthy subjects were recruited from each study site. The mean levels of IL-6 (p < 0.03) and TNF-α (p < 0.01) were significantly different among the study groups. In the case of IL-6, patients from Lima had a mean level of 16.2 pg/ml (healthy) and 48.3 pg/ml (COVID-19), meanwhile, patients from Huaraz had levels of 67.3 pg/ml (healthy) and 97.9 pg/ml (COVID-19). Regarding TNF-α, patients from Lima had a mean level of 25.9 pg/ml (healthy) and 61.6 pg/ml (COVID-19), meanwhile, patients from Huaraz had levels of 89.0 pg/ml (healthy) and 120.6 pg/ml (COVID-19). The levels of IL-2, IL-10 and IFN-γ were not significantly different in the study groups.

**Conclusion:**

Patients with COVID-19 residing at high-altitude tend to have higher levels of inflammatory cytokines compared to patients living at sea level, particularly IL-6 and TNF-α. A better understanding of the inflammatory response in different populations can contribute to the implementation of therapeutic and preventive approaches. Further studies evaluating more patients, a greater variety of cytokines and their clinical impact are required.

## Background

At the end of 2019, a novel coronavirus denominated SARS-CoV-2 rapidly spread through the world causing the pandemic coronavirus disease known as COVID-19 [1]. The majority of patients with COVID-19 are asymptomatic or experienced mild respiratory illness [2]. However, some patients develop a more aggressive disease characterized by fulminant sepsis and acute respiratory failure [2, 3]. Critical and severe infection by SARS-CoV-2, as well as previous coronaviruses, have been linked to an exaggerated inflammatory process denominated “cytokine storm” [4–7]. Under physiological circumstances, a coordinated innate immune response is the first line of defense against viruses; however, a deregulated inflammatory process may cause injury on the host [5]. COVID-19 has been associated with an exuberant activation of the host immune system and the excessive production of proinflammatory cytokines, such as IL-1, IL-2, IL-6, IL-10, IL-12, IFN-γ, TNF-α, among others, which may cause tissue injury, particularly on the lungs [5–7].

High-altitude has been proposed as a protective factor against COVID-19, as lower incidence and fatality rate have been reported in high-altitude areas compared to sea level locations [8–10]. Moreover, these observations have been linked to a decrease of angiotensin converting enzyme 2 (ACE2) in people living at high-altitude, an important trans-membrane protein used by SARS-CoV-2 to infect cells [8]. However, several confounding factors could alter the variability in populations of different geographic areas [11]. One important variable not yet studied, is the difference in the inflammatory response against SARS-CoV-2 infection among people living at different altitudes. Given that chronic hypoxia promotes cytokines production in the absence of disease [12, 13], inflammatory response to SARS-CoV-2 may also be different in these populations. Therefore, the aim of this study was to compare pro-inflammatory cytokines levels among healthy and COVID-19 patients living at different altitudes above sea level.

## Methods

### Study location

A descriptive cross-sectional study was performed in two Peruvian cities at different altitudes for comparison: Lima and Huaraz. Firstly, Lima, which is the capital of Peru, located in the coastal area and at sea level. Lima has a population of 8,574,974, with most of the people living in urban areas. Also, Huaraz is an Andean city located in the region of Ancash, with an altitude of approximately 3052 m above sea level. Ancash has a population of 1,083,519, with most of the people living in rural areas.

### Study subjects

Patients hospitalized in healthcare centers and healthy subjects from Lima and Huaraz were enrolled for comparison of cytokines levels. Participants had to be long term residents in their respective city of study, defined as living in the location for at least 1 year. We included patients hospitalized in COVID-19 wards with mild or moderate disease as defined by Peruvian national guidelines. Mild cases included patients presenting with 2 or more of the following symptoms: cough, fever, rhinorrhea, malaise, sore throat and no severe respiratory distress. Moderate cases included patients with 2 or more of the following symptoms: dyspnea, respiratory rate of > 22, oxygen saturation < 95%, chest X-ray compatible with pneumonia, mental state alteration or hypotension.

For the control group, healthy subjects from each study site who tested negative for SARS-CoV-2 were recruited. Patients from the disease group and control group were matched by age and gender.

Exclusion criteria were patients with previous chronic pulmonary diseases, autoimmune diseases, immunodeficiencies, pregnant women and patients taking immunosuppressant drugs.

### Ethics statement

The study protocol was approved by the Research Ethics Board of the *Instituto de Investigación Nutricional*, Lima, Peru (N° 395-2020/CIEI-IIN). The samples were obtained in the context of the epidemiological/syndromic surveillance program according to the health directives of the National Center for Epidemiology, Disease Control and Prevention of the Ministry of Health of Peru. All methods were performed in accordance with the relevant guidelines and regulation. All the subjects signed a written informed consent for the detection of SARS-CoV-2 in nasopharyngeal samples and allowed the use of the samples for other procedures.

### Diagnosis of COVID-19

A total of 35 COVID-19 patients and 10 healthy subjects were recruited from each study site. Patients were diagnosed with COVID-19 according to Peruvian national guidelines. Patients were considered with a presumptive diagnosis of COVID-19 if attended with an acute respiratory infection presenting cough, sore throat and at least one of the following symptoms: malaise, fever, headache, dyspnea, rhinorrhea or patients with respiratory distress requiring hospitalization. A RT-PCR test from nasopharyngeal samples was used for the detection of SARS-CoV-2 was carried out on patients with suspected infection.

### Quantification of cytokines

Five important proinflammatory cytokines were measured including: IL-6, IL-2, IL-10, IFN-**γ** and TNF-α. Levels of cytokines were quantified using High Sensitivity Human ELISA Kit corresponding for each cytokine (Abcam, United State). Each serum sample was run in duplicate, in accordance with the manufacturer’s instructions.

### Statistical analysis

Cytokines levels were calculated and their mean, maximum and minimum level were reported according to each study group. The difference between means was compared using the ANOVA and post-hoc Turkey Test, a value of p < 0.05 was considered significant. Cytokine levels were divided into three quartiles and categorized into 4 ranges. Percentages were calculated according to each quartile of cytokine level and stacked graphs were created using the software GraphPad Prism 9 (San Diego, CA).

## Results

A total of 35 COVID-19 patients and 10 healthy subjects were recruited from each study site. Cytokine levels were measured (IL-6, IL-10, IL-2, IFN-**γ** and TNF-α) and the minimum, maximum and mean of each group is shown in Table 1. It can be observed that the mean levels of IL-6 (p < 0.04) and TNF-α (p < 0.01) were significantly different among the study groups. A comparison of the inflammatory cytokines in healthy subjects from the two study sites, showed significant differences only in levels of TNF-α (p < 0.01). In the case of IL-6, patients from Lima had a mean level of 16.2 pg/ml (healthy) and 48.3 pg/ml (COVID-19), meanwhile, patients from Huaraz had levels of 67.3 pg/ml (healthy) and 97.9 pg/ml (COVID-19). Regarding TNF-α, patients from Lima had a mean level of 25.9 pg/ml (healthy) and 61.6 pg/ml (COVID-19), meanwhile, patients from Huaraz had levels of 89.0 pg/ml (healthy) and 120.6 pg/ml (COVID-19). The mean levels of IL-2, IL-10 and IFN-γ were not significantly different among groups. Although not significant, it can be observed that patients from Huaraz have greater means of IL-10 and IFN-**γ** compared to patients from Lima, regardless of their health status.

**Table 1:** Maximum, minimum and mean values of cytokines (pg/ml) according to site of study

pg/ml		Lima		Huaraz		P-value*
		Healthy	COVID-19	Healthy	COVID-19	
IL-6	Max	92.8	606.8	372.1	606.8	
	Min	2.4	0.1	1.2	1.2	0.04
	Mean	16.2	48.3	67.3	97.9	
IL-2	Max	28.2	3.8	3.4	53.3	
	Min	1.0	1.0	1.2	1.0	
	Mean	6.5	1.8	2.2	3.3	0.21
IL-10	Max	38.8	153.8	103.6	116.4	
	Min	0.7	0.5	2.2	2.4	
	Mean	9.7	9.0	19.7	15.2	0.53
IFN-γ	Max	76.3	97.4	72.1	97.4	
	Min	4.3	1.0	14.3	5.2	
	Mean	31.7	22.3	34.7	37.5	0.07
TNF-α	Max	50.5	320.8	147.8	288.4	
	Min	7.3	7.3	50.5	28.9	
	Mean	25.9	61.6	89.0	120.6	< 0.01

^*^ANOVA and post-hoc Turkey test were used to calculate p value among means. p < 0.05 was considered significant

**Fig. 1:**
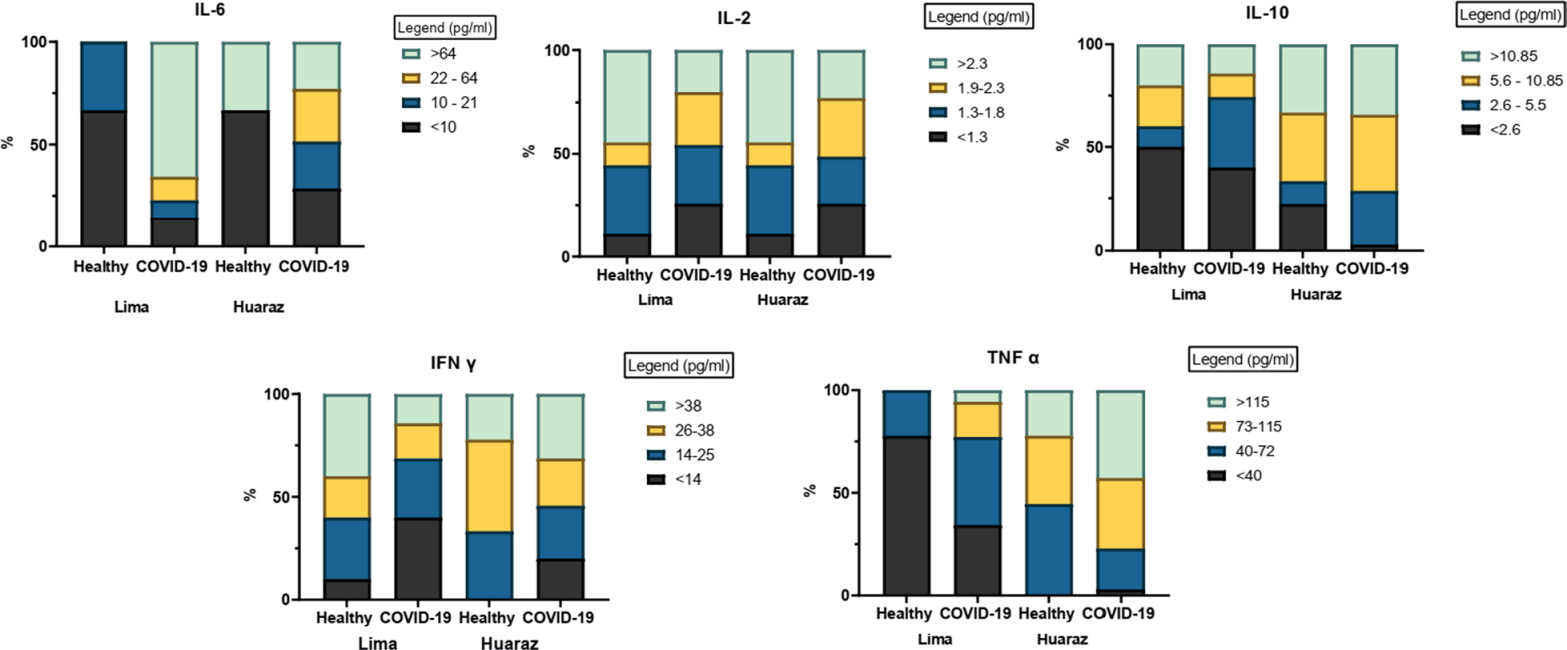
Ranges of cytokines levels divided into 4 ranges, according to study group

Figure 1 shows the calculated percentage of patients corresponding to each range of cytokines levels. A total of four ranges were created according to three quartiles for each cytokine. Ranges were calculated in pg/ml as follows: for IL-6 (< 10, 10–21, 22–64 and > 64), for IL-2 (< 1.3, 1.3–1.8, 1.9–2.3 and > 2.3), for IL-10 (< 2.6, 2.6–5.5, 5.6–10.85 and > 10.85), for IFN-γ (< 14, 14–25, 26–38 and > 38) and for TNF-α (< 40, 40–72, 73–115, > 115).

It can be observed that in the case of IL-6, IL-10 and TNF-α, a greater proportion of COVID-19 and patients from Huaraz corresponded to the upper quartiles compared to Lima patients. For example, for IL-6 more than 50% of the healthy patients in Lima and Huaraz show levels below the first quartile (10 pg/ml), on the contrary, COVID-19 patients show greater percentage in upper values. In the case of TNF-α, a clear tendency can be observed, with more healthy subjects from Lima showing values in the lower quartiles, while most of the healthy and COVID-19 patients from Huaraz are categorized in the upper ranges. A clear pattern in the graphs of IL-2 and IFN-γ could not be observed, as large heterogeneity was observed among groups.

## Discussion

COVID-19 is a complex and heterogeneous disease caused by SARS-CoV-2. Its clinical presentation, severity and prognosis are highly variable and depend on several factors. Two of the most important variables with probable clinical impact are the levels pro-inflammatory cytokines levels and the residing altitude of the patients. Currently, there are approximately 400 million people who are permanently living at high altitude, which is considered at elevations higher than 1500 m above sea level [14]. The high-altitude environment comprises several conditions that can potentially modify morbidity and mortality [14–16]. Since the beginning of the pandemic, it has been hypothesized, based on incidence and mortality records, that high-altitude may be a protective factor against SARS-CoV-2 transmission and disease severity [9, 10]. A plausible biological mechanism that could explain this finding is the lesser expression of angiotensin-converting enzyme-2 (ACE-2) in residents of high altitude [8]. Moreover, an interaction between ACE-2 and inflammatory cytokines have been proposed. ACE-2 is an important component of the renin-angiotensin system (RAS), which also plays a pivotal role in inflammation and pathological changes in lung injury [17, 18]. Also, it has been proposed that ACE-2 might have a role in the overexpression of cytokines such as IL-6, IL-7, TNF-α, IL-2, IL-1β, among others [17].

However, the interplay between high altitude and inflammation has not been addressed in the context of COVID-19, we consider that this three-way interaction may have important clinical implications [19]. Mainly because high altitude and chronic hypoxia induce a permanent state of inflammation among healthy people residing in those areas. It has been demonstrated that exposure to chronic hypoxia promotes the nuclear factor, which stimulates the production of several pro-inflammatory cytokines including IL-6, IL-10, TNF-α, among others [12, 15, 16]. In general, molecular pathways involving inflammation, infection and hypoxia are likely to be linked [16]. Therefore, we performed the first study evaluating the differences in the cytokine levels among patients with COVID-19 and healthy subjects, in relation to the altitude in which they reside. The evaluation of 5 important pro-inflammatory cytokines was performed, these molecules were chosen based on previous reports on their importance on COVID-19 infection and clinical severity [5–7, 15].

Our analysis show that the mean levels of IL-6 and TNF-α were significantly different in the study groups, with higher levels in COVID-19 patients and those residing at high altitude. Levels of IL-2, IL-10 and IFN-γ did not show a significant difference among groups, however, when comparing the study groups, COVID-19 patients in high altitude showed a tendency to present high levels of these cytokines. These findings are in agreement with previous reports of studies evaluating cytokines levels in COVID-19 patients living at sea level. For example, Yang et al. [20] determined that more than 30 cytokines can be significantly elevated in patients with COVID-19, of which, 14 could be associated with disease prognosis and severity prediction. Another study performed by Han et al. [7]. demonstrated that levels of IL-6, IL-2, IL-10, IFN-γ and TNF-α were higher in COVID-19 patients than in healthy subjects. Among these cytokines, the authors found that IL-6 and IL-10 were independent factors for disease severity. One of the largest cohorts of patients carried out by Del Valle et al. [6]. reported that IL-6 and TNF-α were strong disease predictors and independent factors associated with death, proposing that therapeutic agents should target these biomolecules.

There are multiple studies reporting the association between different cytokines and COVID-19 disease [21–23], however, no previous studies have been carried out comparing the levels of these molecules in people residing at different altitudes. This is the first exploratory study that reports differences in the average and percentage of patients with high levels of cytokines, according to the altitude. Our findings indicate a greater inflammatory response in patients living at high altitude compared to sea level, however, COVID-19 occurrence and mortality is reported to be lower in patients at higher altitudes [24]. We propose some mechanisms that may explain this finding. As mentioned before, patients residing at high altitude are chronically exposed to slightly elevated levels of inflammatory factors, which consequently leads to adaptation and immune tolerance [25]. When infected by the virus, these patients may develop a less severe disease as their body is already adapted to elevated levels of inflammatory factors. Another proposed mechanism are the polymorphisms of the ACE 2 gene in populations at high altitude. The low ACE2 activity may protect them against the infection and replication of the virus [26]. The interaction between inflammatory cytokines and ACE 2 levels could have an important role in disease severity and should be further studied.

On the other hand, other factors apart from inflammation may explain the better outcomes in patients at high altitude. Environmental conditions related to barometric pressure, oxygen availability and UV radiation may also have a role in disease severity. Finally, physiologic adaptations in these subjects could explain the better outcomes in these patients as they have a better tolerance to hypoxia [26, 27]. The understanding of these variables could contribute to a better understanding of the pathogenesis of COVID-19 in these populations and consider therapeutic and preventive measures.

## Conclusion

Our results evidence that patients with COVID-19 residing in high-altitude tend to have higher levels of inflammatory cytokines, particularly IL-6, IL-10 and TNF-α.

These findings contribute to the understanding of the response to COVID-19 infection in people residing at different altitudes. Other factors should also be evaluated in these populations, as chronic hypoxia may contribute to different physiological adaptations.

A better understanding of the inflammatory response in different populations can contribute to the implementation of therapeutic and preventive approaches. Further studies evaluating more patients, a greater variety of cytokines and their clinical impact are required.

### Limitations

Firstly, as this was a first exploratory analysis on the subject, the sample size was limited. Also, a semi quantitative method to measure cytokine level was used, such as ELISA. The association between clinical characteristics and cytokines levels was not evaluated in the current study, given our study design and limitations. Another important limitation is that levels of ACE-2 were not measured, which could have an impact on the expression of inflammatory cytokines. Finally, follow-up was not performed, and it would be interesting to evaluate the changes of cytokines according to day of infection and post-infection.

## Data Availability

The datasets generated and/or analysed during the current study are available in the figshare repository, LINK: https://figshare.com/articles/dataset/Dataset_SARS-CoV2-cytokines_y2021m02_50_/14050610

## Abbreviations

ACE2: Angiotensin converting enzyme 2
ICU: Intensive care unit
IL-6: Interleukin-6
IL-2: Interleukin-2
IL-10: Interleukin-10
IFN-γ: Interferon gamma
TNF-α: Tumor necrosis factor alpha
RT-PCR: Reverse transcription polymerase chain reaction
